# Related Structure Characters and Stability of Structural Defects in a Metallic Glass

**DOI:** 10.3390/ma11040468

**Published:** 2018-03-22

**Authors:** Xiaofeng Niu, Shidong Feng, Shaopeng Pan

**Affiliations:** 1 College of Materials Science and Engineering, Taiyuan University of Technology, Taiyuan 030024, China; niu.xiao.feng@126.com; 2 Shanxi Key Laboratory of Advanced Magnesium-Based Materials, Taiyuan University of Technology, Taiyuan 030024, China; 3 Advanced Manufacturing Technology Research Centre, Department of Industrial and Systems Engineering, Hong Kong Polytechnic University, Hong Kong 999077, China; shidong.feng@polyu.edu.hk

**Keywords:** metallic glass, quasi-nearest atom, molecular dynamics simulations

## Abstract

Structural defects were investigated by a recently proposed structural parameter, quasi-nearest atom (QNA), in a modeled Zr_50_Cu_50_ metallic glass through molecular dynamics simulations. More QNAs around an atom usually means that more defects are located near the atom. Structural analysis reveals that the spatial distribution of the numbers of QNAs displays to be clearly heterogeneous. Furthermore, QNA is closely correlated with cluster connections, especially four-atom cluster connections. Atoms with larger coordination numbers usually have less QNAs. When two atoms have the same coordination number, the atom with larger five-fold symmetry has less QNAs. The number of QNAs around an atom changes rather frequently and the change of QNAs might be correlated with the fast relaxation metallic glasses.

## 1. Introduction

Although metallic glasses (MGs) have received much attention since its discovery in 1960s due to their outstanding mechanical properties such as ultrahigh strength, superior elasticity, and excellent thermo-plasticity, some weaknesses limit their industrial applications, including the difficulty to obtain normal size and brittleness at room temperatures [1,2,3,4]. The latter weakness makes MGs impossible to be used as structural materials. It is worth noting that sub-micrometer-scale MGs can exhibit large plastic deformation together with a large yield strength [5,6]. Therefore, how to overcome this weakness is the key point for the massive industrial applications of MGs. Full understanding the deformation mechanism of MGs is helpful to solve this problem [7]. In crystals, the structural defects such as dislocation play a key role in the deformation of crystalline materials. However, it is difficult to characterize structural ‘defects’ in MGs because the atomic structures of MGs are disordered in the long range [8,9].

The term of free volume was used as structural ‘defects’ to study the deformation in MGs in many previous works [10,11,12,13]. Although structural defects in MGs might be located near free volume, the number of structural defects in MGs is not equivalent to the total free volume. One simple example is that the number of structural defects increases under a compressive deformation while the free volume decreases. Other structural parameters, such as localized soft mode and local fivefold symmetry, play an important role in structure–property relationship in metallic liquids and MGs [14,15]. However, localized soft mode cannot provide a clear picture of local atomic structure. While local five-fold symmetry has some correlation with free volume, it only reflects the local geometry of atomic packing in MGs. Therefore, they cannot be used to characterize the structural defects in MGs. To solve this problem, recently the term of flow unit has been frequently used to character the structure ‘defects’ in MGs [16,17]. Flow units refer to the regions where the atomic packing is quite loose in MGs. Thus, to search flow units in MGs, a structure parameter is needed to effectively identify the degree of atomic packing.

In previous works, we proposed a new structural parameter, quasi-nearest atom (QNA), and found that QNA is closely correlated with dynamic heterogeneity in metallic liquids and also with mechanical heterogeneity in MGs [18,19,20,21]. We think that QNA is a useful tool to describe structure ‘defects’ in metallic liquids and glasses. In this work, we will investigate the basic features of structural ‘defects’ by QNA in a modeled Zr_50_Cu_50_ MG.

## 2. Materials and Methods

Classical molecular dynamics (MD) simulations were performed to investigate the structural defects in a model Zr_50_Cu_50_ MG containing 100,000 atoms that interact with an embedded-atom method (EAM) potential [22]. For the sample preparation, the system was equilibrated at *T* = 2000 K for enough time to make sure that the potential energy keeps dynamically equilibrated. Then the configuration was cooled down to *T* = 300 K with a cooling rate of 10^12^ K/s and relaxed for 1 ns at300 K to obtain the MG sample. The cell size was adjusted to give zero pressure during the sample preparations with the constant number, pressure, and temperature (NPT) ensemble. Periodic boundary conditions (PBCs) were applied in all the three dimensions. Voronoi tessellation method was carried out to describe the local structures in the MG [23]. Before Voronoi construction, a program for simplification was performed as follows. Supporting that atoms A and B are two neighbors of atom O, if $\vec{OA}$·$\vec{OB}$ > |$\vec{OB}$|^2^, atom A will not be considered to be the nearest neighbor of atom O. Further analysis based on Voronoi analysis was performed to calculate the number of quasi-nearest atoms (QNAs) for each atom [16]. If two atoms are identified as a pair of QNAs, they should satisfy three conditions: (1) they share a common nearest neighbor; (2) their corresponding Voronoi faces of the Voronoi polyhedron centered by their common nearest neighbor share an edge; and (3) they are not the nearest neighbors of each other. As pointed in our previous work, there is a large free volume between a pair of QNAs. Therefore, QNA can be used to describe the structural defects in metallic glasses.

## 3. Results and Discussion

**Figure 1:**
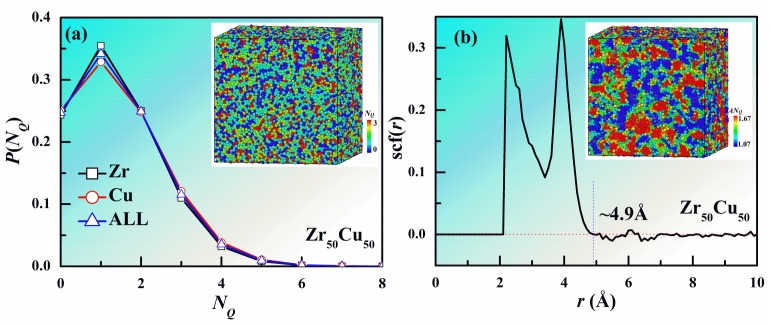
Quantitative and spatial distribution of *N_Q_* in Zr_50_Cu_50_ MG at 300 K. (**a**) Quantitative distribution. Inset is a configuration with atoms colored by different *N_Q_*. (**b**) Spatial correlation function (scf(*r*)). The spatial correlation length for *N_Q_* is about 4.9 Å. we calculated *AN_Q_* for each atom as the average *N_Q_* value of itself and its neighboring atoms with a distance less than 4.9 Å. Inset is the same configuration in (**a**) but with atoms colored by different *AN_Q_*.

Firstly, we present the quantitative and spatial distribution of the number of QNAs (*N_Q_*) in Zr_50_Cu_50_ MG at 300 K in Figure 1. The quantitative distributions of *N_Q_* around Cu and Zr follow a similar behavior as shown in Figure 1a. However, the fraction of Cu atoms with small *N_Q_* is slightly smaller than that of Zr, and the situation is opposite for the atoms with large *N_Q_*. This indicates that the local packing of Cu is a little looser than that of Zr. Because of inherent limitations of MD method, the time scale in MD simulation is much shorter than experimental time scale. The local order of MGs can be significantly different with different cooling rates. The effects of different strain rates are beyond the scope of the present study, and hence we focus our discussions below on the same cooling rate. We note that the fraction shows a peak at *N_Q_* ~ 1 and only about 25% of the atoms have no QNA, suggesting that most of the atoms are not closely packed in the MG. Figure 1a also shows a configuration with atoms colored by their *N_Q_*. It can be clearly seen that the atoms with more QNAs intend to aggregate together, indicating the spatial heterogeneities of QNA. To directly reflect the spatial heterogeneities of QNA, we investigated the space correlation function for NQ which is defined as

(1) $$\mathrm{scf}\left(r\right)=\frac{1}{N}\sum_{i=1}^{N}\frac{\langle \left(N_{Q}\left(i\right)-\langle N_{Q}\rangle \right)\left(\langle N_{Q}\left(i,r,r+dr\right)\rangle -\langle N_{Q}\rangle \right)\rangle }{D{\left(N_{Q}\right)}^{2}}$$

where *N_Q_*(*i*) is the value of *N_Q_* for atom *i*, <*N_Q_*(*i, r, r + dr*)> is the average value of *N_Q_* for atoms with a distance to atom *i* within *r* and *r + dr*, and <*N_Q_*> and *D*(*N_Q_*) are the average value and standard deviation of *N_Q_* for the system. The value of scf(*r*) is within 1 and −1. The larger the absolute value of scf(*r*) is, the larger space correlation for *N_Q_* is. When scf(*r*) is near to 0, there is almost no space correlation for *N_Q_*. Figure 1b displays the space correlation function for *N_Q_*. When the distance r is less than about 2.1 Å, the value of scf(*r*) is 0, because the distance between two atoms in the system is larger than that one. As r increases, the value of scf(*r*) increases rapidly and then decays to around 0 beyond 4.9 Å, which indicating that there is almost no space correlation for *N_Q_* beyond 4.9 Å. Therefore, the space correlation length (SCL) for *N_Q_* can be regarded as 4.9 Å. To better reflect the spatial heterogeneities of QNA, we have coarse-grained *N_Q_* by assigning to each atom the value of *N_Q_* (namely *AN_Q_*) averaged over the local values for that atom and of the atoms lying within a distance *r* = SCL of that atom. We calculated the coarse-grained *N_Q_* (*AN_Q_*) for each atom and colored them with it as shown in the inset of Figure 1b. It can be seen that structure heterogeneity reflected by *AN_Q_* is much clearer than that by *N_Q_*.

**Figure 2:**
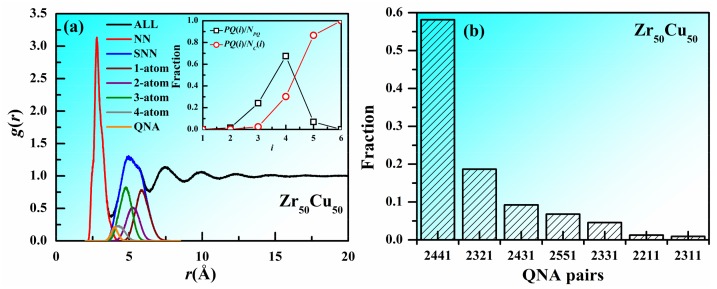
Correlation between QNA and cluster connection in Zr_50_Cu_50_ MG at 300 K. (**a**) pair distribution function g(r) for Zr_50_Cu_50_ MG at 300 K. The decomposed pair distribution function for the nearest neighbors (NN), second nearest neighbors (SNN) via one-atom, two-atom, three-atom, and four-atom cluster connections and QNAs, are also included. Insets are the fractions of *PQ*(*i*)/*N_PQ_* and *PQ*(*i*)/*Nc*(*i*) as a function of the number of shared atoms (*i*) in cluster connections. *PQ*(*i*) is the number of QNA pairs with i-atom cluster connection, *N_PQ_* is the number of all the QNA pairs and *Nc*(*i*) is the number of *i*-atom cluster connections. (**b**) Distribution of different types of QNA pairs.

An atom and its nearest neighbors (NN) are often regarded to be in a cluster and if two atoms share common nearest neighbors, the clusters centered by the two atoms are regarded to be connected. Especially if the central atoms of two connected clusters are not the nearest neighbors to each other, the cluster connection is thought to be non-interpenetrating connections. The non-interpenetrating connected clusters usually share one, two, three, or four atoms, which are denoted hereafter as one-atom, two-atom, three-atom, and four-atom connections, respectively. According to the definition of QNA, a pair of QNAs are not the nearest neighbors to each other and share at least one common nearest neighbor. This fact indicates that the clusters centered by a pair of QNAs are non-interpenetrating connected and there is close correlation between QNA and cluster connection in MGs. Figure 2a shows the decomposed pair distribution function in Zr_50_Cu_50_ MG at 300 K. It can be seen that the nearest neighbors determined by Voronoi analysis and the central atoms of the non-interpenetrating connected clusters can perfectly construct the first and the second peaks of the pair distribution function, respectively. The decomposed pair distribution function for second nearest neighbors (SNN) via one-atom, two-atom, three-atom, and four-atom cluster connections and QNAs, are also included. It can be seen that the curve for QNAs has a peak near the first minimum of pair distribution function, indicating the shorter distance between a pair of QNAs than most of the second nearest neighbors. The curve for QNAs has much large overlap with those for three-atom and four-atom cluster connections, small overlap with that two-atom cluster connection and almost no overlap with that for one-atom cluster connection, confirming the close correlation between QNA and cluster connection. Figure 2a also displays the fractions of *PQ*(*i*)/*N_PQ_* and *PQ*(*i*)/*Nc*(*i*) as a function of the number of shared atoms (*i*) in cluster connections. Here, *PQ*(*i*) is the number of QNA pairs with i-atom cluster connection, *N_PQ_* is the number of all the QNA pairs and *Nc*(*i*) is the number of i-atom cluster connections. It can be seen that two clusters centered by a pair of QNAs are usually three-atom and four-atom connected and as the number of shared atoms increases, the fraction of QNAs in the cluster connections increases. To better describe the correlation between QNA and cluster connections, we employed the bond pair analysis technique proposed by Honeycutt and Anderson. The bond pairs are expressed as *i*, *j*, *k*, and *l*. When two atoms are the nearest neighbors of each other, the two atoms are called a bond pair, with *i* = 1, otherwise *i* = 2; *j* denotes the atom number of bonds forming with both the two atoms; *k* is the bond number among the above j atoms; and *l* is used to distinguish the local structures when *k* is the same. Figure 2b shows the distribution of different types of QNA pairs. It can be seen that the amount of 2441 is much larger than the other QNA pairs. The second- and third-largest number of QNA pairs are 2321 and 2431, which both have the index *j* larger than *k*. All these facts suggest that there is close correlation between QNA and cluster connection in MGs.

**Figure 3:**
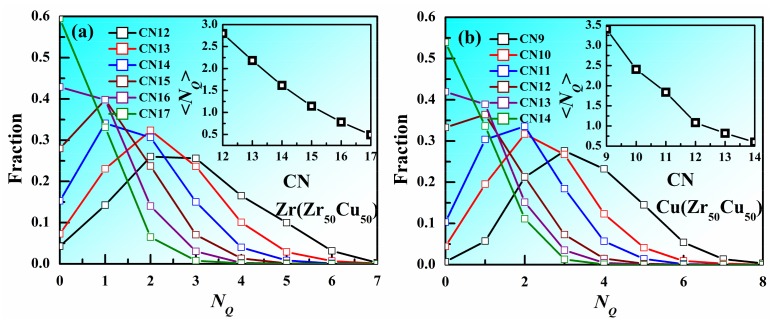
Distribution of *N_Q_* for the atoms with different CNs in Zr_50_Cu_50_ MG at 300 K. (**a**) Zr, (**b**) Cu. Inset is the average *N_Q_* as a function of CN.

Voronoi tessellation method is one of the tools used most frequently to describe the local structure of metallic liquids and glasses. A plane is drawn to bisect each line connecting the center atom and one of the neighboring atoms, and the polyhedron enclosed by all the inner planes is called a Voronoi polyhedron (VP). The VP can be labeled by the Voronoi index <*n*_3_, *n*_4_, *n*_5_, *n*_6_>, where *n_i_* denotes the number of *i*-edged faces of the VP, to describe the arrangement and symmetry of the nearest-neighbor atoms around the center atom. The coordination number (CN) concept is employed to characterize the number of nearest neighbors which can be determined by Voronoi method (CN = ∑*n_i_*). If the CN of an atom is much larger than that of another atom with the same element type, the atomic packing around this atom might be thought to be much denser. Therefore, does larger CN corresponds to denser atomic packing? Figure 3a displays of *N_Q_* for the atoms with different CNs in Zr_50_Cu_50_ MG at 300 K at 300 K. The distribution of *N_Q_* for Zr atoms with CN = 12 in Zr_50_Cu_50_ has a peak at *N_Q_* 2~3. As CN increases, the peak moves to smaller *N_Q_*, indicating the atomic packing of atoms with larger CN tend to be denser. However, it should be noted that the distributions with different CN have overlapping, suggesting that the atomic packing of some atoms with larger CN might be loose. The distribution of *N_Q_* for Cu atoms with different CN shown in Figure 3b also displays the similar trend. Insets of Figure 3 show that, as CN increases, the average *N_Q_* decreases for both Zr and Cu atoms. These facts indicate that larger CN are helpful to reduce *N_Q_*, but not decisive.

**Figure 4:**
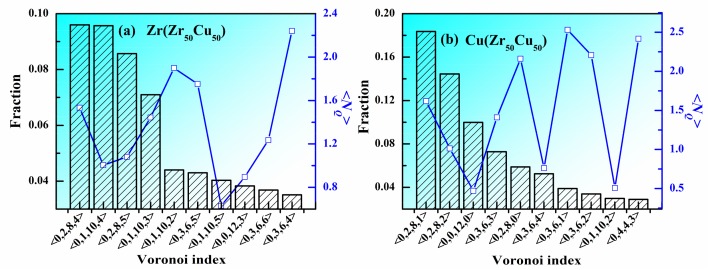
Fraction and <*N_Q_*> of top 10 Voronoipolyhedra in Zr_50_Cu_50_ MG at 300 K. (**a**) Zr, (**b**) Cu.

Besides the CN, do other structural features influence the atomic packing in MGs? In many MGs, some favored VPs are considered as the structural units to construct the atomic structure of the whole system. Figure 4a,b show the fraction and <*N_Q_*> of top 10 VPs around Zr and Cu in Zr_50_Cu_50_ MG at 300 K. Around Zr atoms, the <0,2,8,4> VP has the largest population but its <*N_Q_*> is not the lowest one, indicating that its atomic packing is not the densest. All the 10 top VPs can be classified into several groups according to their CNs. It can be seen that the VPs with CN = 16 (<0,1,10,5>) have the lowest <*N_Q_*>, the VPs with CN = 15 (<0,0,12,3>, <0,1,10,4>, <0,2,8,5> and <0,3,6,6>) have the second lowest <*N_Q_*>, the VPs with CN = 14 (<0,1,10,2>, <0,2,8,4> and <0,3,6,5>) have the third lowest <*N_Q_*> and the VPs with CN = 13 (<0,1,10,2> and <0,3,6,4>) have the largest <*N_Q_*>. These facts are consistent with the conclusion obtained by Figure 3, which is that larger coordination numbers correspond to lower <*N_Q_*>. For a certain CN, take CN = 15 for an example. The order is <*N_Q_*>(<0,0,12,3>) < <*N_Q_*>(<0,1,10,4>) < <*N_Q_*>(<0,2,8,5>) < <*N_Q_*>(<0,3,6,6>). Therefore, larger five-fold symmetry (*n*_5_) corresponds lower <*N_Q_*>. The situations for CN = 14 and CN = 13 are the same to that for CN = 15. Around Cu atoms, the <0,2,8,1> VP has the largest population but its <*N_Q_*> is not the lowest one, indicating that its atomic packing is not the densest. The main CNs around Cu atoms are 10, 11, 12, and 13. Different from Zr atoms, although the <0,1,10,2> and <0,3,6,4> VPs has the largest CN (13), they do not have the lowest <*N_Q_*>. The <0,0,12,0> VP, well known as icosahedral polyhedron, has the lowest <*N_Q_*>, indicating that its atomic packing is rather dense, which might be the reason why icosahedral clusters play an important role in the stability of liquid structure. For a certain CN, the conclusion that larger five-fold symmetry corresponds lower <*N_Q_*> also holds around Cu. For example, For CN = 12, <*N_Q_*> (<0,0,12,0>) < <*N_Q_*>(<0,2,8,2>) < <*N_Q_*>(<0,3,6,3>) < <*N_Q_*>(<0,4,4,4>). Therefore, besides coordination number, five-fold symmetry also plays an important role in the atomic packing in MGs.

**Figure 5:**
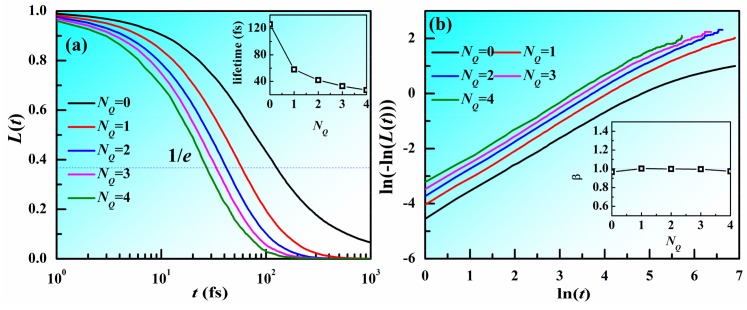
Lifetime for atoms with different *N_Q_* in Zr_50_Cu_50_ MG at 300 K. (**a**) Lifetime function (*L*(*t*)) for different *N_Q_* as a function of time *t*. Inset is the lifetime of different *N_Q_*. (**b**) ln(−ln(*L*(*t*))) vs. ln(*t*), inset is the slope with ln(*t*) within [0, 3] as a function of *N_Q_*.

In crystals, structural defects are usually located at the regions with high energy and low stability. In our previous work, it is found that atoms with larger *N_Q_* have high potential energy and faster atomic mobility in metallic liquids. The atomic structure in MGs at room temperatures is usually considered to be almost unchanged. Therefore, does the value of *N_Q_* around an atom have a long lifetime? Figure 5 displays the lifetime function (*L*(*t*)) for different *N_Q_* which is defined as the fraction of atoms with *N_Q_* unchanged during the time range [0, *t*] as a function of the time t. The time resolution for the investigation is as long as the timestep, 1 fs. It can be seen that, as time increases, *L*(*t*) rapidly decays. As *N_Q_* increases, *L*(*t*) with small *N_Q_* decays more slowly compared to that with larger *N_Q_*. This indicates that atoms with smaller *N_Q_* tend to change slower than those with larger *N_Q_*. The lifetime is defined as the time at which the *L*(*t*) decays to 1/e of its initial value. As shown in the inset of Figure 5a, the lifetime decreases with increasing *N_Q_*. It should be noted that the lifetimes for different *N_Q_* are rather short, suggesting that the value of *N_Q_* around an atom changes quite frequently. Figure 5b shows ln(−ln(*L*(*t*))) vs. ln(*t*). The curves display a linear correlation. We plotted their slope β in the inset. It can be seen that β is around 1 for all the *N_Q_*, indicating that *L*(*t*) satisfies the exponential relation as follows: *L*(*t*) = e^−*t*/τ^. The change of *N_Q_* might be correlated with the fast relaxation in MGs.

## 4. Conclusions

In this work, we study the basic features of structural ‘defects’ in a modeled Zr_50_Cu_50_ MG by molecular dynamics (MD) simulations. By calculating the number of QNAs (*N_Q_*) for each atom, we can quantify the degree of structural ‘defects’ of an individual atom. From the present analysis, *N_Q_* exhibits significant spatial heterogeneities in the MG. The QNA shows close correlation with other structural parameters, such as cluster connection, coordination number, and five-fold symmetry. Most of QNA pairs are the center atoms of the clusters with four-atom connections. Larger coordination number usually corresponds to smaller *N_Q_*. When two atoms have the same coordination number, the atom with larger five-fold symmtry has a smaller *N_Q_*. Meanwhile, the value of *N_Q_* around an atom changes rather frequently. The lifetime function for different *N_Q_* satisfies an exponential relation, indicating that the change of *N_Q_* might be correlated with the fast relaxation in MGs. We think that QNA is a useful tool to character the local order in MGs including normal bulk samples and also thin films. More work will be done to investigate the structure–property relationship in these MG systems in the future.
